# Characterizing conjugative plasmids from an antibiotic-resistant dataset for use as broad-host delivery vectors

**DOI:** 10.3389/fmicb.2023.1199640

**Published:** 2023-06-14

**Authors:** Héctor G. Loyola Irizarry, Ilana L. Brito

**Affiliations:** Meinig School of Biomedical Engineering, Cornell University, Ithaca, NY, United States

**Keywords:** plasmids, conjugation, broad host-range (BHR), CDC & FDA AR Isolate Bank, microbiome engineering

## Abstract

Human microbiome engineering is increasingly proposed as a way to modulate health outcomes. However, one of the current limitations to engineering microbial communities *in situ* is delivery of a genetic payload for introducing or modifying genes. Indeed, there is a need to identify novel broad-host delivery vectors for microbiome engineering. Therefore, in this study, we characterized conjugative plasmids from a publicly available dataset of antibiotic-resistant isolate genomes in order to identify potential broad-host vectors for further applications. From the 199 closed genomes available in the CDC & FDA AR Isolate Bank, we identified 439 plasmids, of which 126 were predicted to be mobilizable and 206 conjugative. Various characteristics of the conjugative plasmids, such as size, replication origin, conjugation machinery, host defense mechanisms, and plasmid stability proteins, were analyzed to determine these plasmids’ potential host-range. Following this analysis, we clustered plasmid sequences and chose 22 unique, broad-host range plasmids that would be suitable for use as delivery vectors. This novel set of plasmids will provide a valuable resource for engineering microbial communities.

## 1. Introduction

Recent advances in our knowledge of host-associated microbiomes have made it clear that these communities are crucial to maintain their host’s health. Changes in the community structure of a microbiome can result in a disease-associated state known as “dysbiosis” (Guirro et al., 2019; Sobhani et al., 2019; Tanja Dapa et al., 2022). It has also been shown that some human gut commensal bacteria perform deleterious functions, contributing to disease (Rath et al., 2018). Microbiome engineering, direct manipulation of the microbial community, has emerged as a way to avoid these negative health outcomes. However, given the level of complexity of microbial communities, altering the community structure makes it difficult to determine the mechanisms that underlie the effects observed when the microbiome is engineered (Alexander and Turnbaugh, 2020). One potential way to manipulate the microbiome without altering community structure is by modifying the genome of existing community members.

Plasmids are extra-chromosomal DNA molecules found mostly in bacteria. These genetic elements can be non-mobile or mobilized between cells through conjugation, one of the primary forms of horizontal gene transfer (HGT) (Smillie et al., 2010). Recently, conjugation has been proposed as a method for synthetic DNA delivery through microbiomes (Ronda et al., 2019; Neil et al., 2021b). This strategy provides a targeted approach, resulting in species- or gene-specific manipulation, which can lead to detectable mechanistic and ecological effects. This lies in contrast to broad methods of microbiome manipulation, such as the use of prebiotics (Enam and Mansell, 2019), use of probiotics (Mao et al., 2018), and fecal microbiota transplant (FMT) (Guirro et al., 2019), which produce global effects in the community whose outcomes might be hard to predict. Additionally, since plasmid transfer has been observed across phyla (Klümper et al., 2015) and even across domains (Bates et al., 1998), conjugation has the potential to deliver DNA to a wide variety of hosts. It has been shown that, in the human gut microbiome, some functions are not limited to a single species (Rath et al., 2017). Therefore, in order to fully eliminate a function from the community, it may be necessary to be able to perform genetic manipulations in various microbes at once.

Current microbiome engineering technologies have been shown to modify single species in the community (Hsu et al., 2020; Lam et al., 2021; Neil et al., 2021b; Jin et al., 2022; Rubin et al., 2022). Many of these methods use phage as a delivery system (Hsu et al., 2020; Lam et al., 2021), which limits their host-range as most phage only infect a single species or strain (Yosef et al., 2015). Most methods that use plasmids as their delivery system are limited either by their conjugation rate (Ronda et al., 2019) or by host-range (Neil et al., 2021b). Finally, methods that use modular plasmids that can be modified depending on their intended target provide the flexibility to target multiple species but are work-intensive due to the need to identify gene transfer methods for potential recipients (Jin et al., 2022; Rubin et al., 2022). Indeed, an all-in-one broad-host delivery plasmid would stream-line microbiome engineering applications, such as the targeted insertion of specific genes or the removal of deleterious functions from the community.

The host-range of a given plasmid is directly influenced by how the plasmid evades the various barriers to DNA transfer in different hosts, which can be determined by both plasmid- and host-encoded factors (Thomas and Nielsen, 2005). During the initiation of conjugation, barriers might arise in the compatibility of a recipient and the donor’s conjugation system (Smillie et al., 2010). Once a plasmid is transferred, it must be able to replicate in its host, leading to another potential barrier (Benz et al., 2021). Aside from replication, the plasmid must also avoid host defenses, such as restriction-modification (R-M) and CRISPR-Cas systems (Bernheim and Sorek, 2020). Finally. plasmid stability is also required in order to be able to be replicated through vertical transmission in a new host (Brockhurst and Harrison, 2022).

Although the plasmid-encoded factors that can affect a given plasmid’s host-range are relatively well understood, it has not been a focus of optimization in microbiome engineering technologies. One method to potentially obtain broad host-range (BHR) plasmids is to search for these in existing datasets, which has the advantage of resulting in vectors that are adapted for survival in a set range of recipients. Therefore, in this study we characterized plasmids from closed genomes in the CDC & FDA Antibiotic Resistance (AR) Isolate Bank and estimated their host-range to identify candidate plasmids that could be used as broad-host delivery vectors. We chose this public set of isolates because conjugative plasmids have been shown to commonly carry AR genes due to the selective advantage these genes provide (Che et al., 2021; Newbury et al., 2022). Additionally, this collection provides a convenient resource for researchers to easily obtain these plasmids and test their host-range. To estimate each plasmid’s host-range, we analyzed plasmid replication genes, conjugation-associated genes, plasmid stability and maintenance genes, and host defense genes. We present a toolbox of 22 plasmids that could potentially be used as delivery vectors with varying host-range.

## 2. Materials and methods

### 2.1. Retrieval of CDC & FDA AR Isolate Bank plasmid sequences

CDC & FDA AR Bank assemblies were downloaded from NCBI (BioProject accession PRJNA294416). Only assemblies with complete genomes were downloaded to ensure quality of putative plasmid sequences. This included sequences from isolates in the following ten panels: “Ceftazidime/avibactam,” “Ceftolozane/tazobactam,” “Enteric Pathogen Diversity,” “Enterobacteriaceae Carbapenem Breakpoint,” “Enterobacteriaceae Carbapenem Diversity,” “Gram Negative Carbapenemase Detection,” “Isolates with New or Novel Antibiotic Resistance,” “*Pseudomonas aeruginosa*,” “Staphylococcus with Borderline Oxacillin Susceptibility,” and “Vancomycin Intermediate *Staphylococcus aureus*.” After assemblies were downloaded, sequences labeled as ‘plasmid’ were used for further analysis.

### 2.2. Identifying replication and host-range associated sequences

Open reading frames (ORFs) for each recovered plasmid sequence were obtained using Prodigal v2.6.3 (Hyatt et al., 2010). A protein database was then created from these ORFs and used for BLASTp v2.13.0 to identify replication, virulence, partitioning, toxin-antitoxin, SOS inhibition and mating-pair stabilization proteins. Rep protein sequences were obtained from NCBI as previously described (Shintani et al., 2015). Virulence protein sequences were obtained from the VFDB (Liu et al., 2022). Toxin-antitoxin sequences were obtained from the TADB (Xie et al., 2018). Due to some antitoxins being RNA opposed to protein, a nucleotide database from the plasmid sequences was created and BLASTn was used to identify the RNA antitoxin sequences. Finally, partitioning, SOS inhibition, and mating-pair stabilization protein sequences were obtained from UniProt by downloading the UniRef50 clusters associated with well-studied proteins associated with these functions (The UniProt Consortium, 2023). Supplementary Table S2 describes all the reference proteins used for these analyses. Replication and toxin-antitoxin protein hits were called with the following parameters: e-value <10^−5^, > 50% identity, and > 50% query coverage as previously described (Shintani et al., 2015). For all other proteins, the threshold of query coverage was raised to >70% as previously described (Shintani et al., 2015). For partitioning, SOS inhibition, and mating-pair stabilization proteins, the percent identity threshold was raised to >80% since the databases used for these proteins included protein sequences that were not experimentally verified. The Comprehensive Antibiotic Resistance Database’s (CARD) Resistance Gene Identifier v5.2.1 was used to identify ARGs in the plasmid sequences (Alcock et al., 2023). Finally, rmsFinder was used to determine if a plasmid contained methyltransferase sequences (Oliveira et al., 2016). True hits were defined as sequences that met rmsFinder’s built-in coverage threshold, an e-value <10^−5^, and > 50% identity.

### 2.3. Determining plasmid mobility

The previously described MOB-Suite v3.1.0 was used to determine the presence of relaxase genes, T4SS genes, and oriT, as well as a prediction of both mobility and host-range (Robertson and Nash, 2018; Robertson et al., 2020). All recovered plasmid sequences were run through MOB-Suite’s “MOB-typer” tool with its default settings and results for relaxase and T4SS genes were verified by BLASTp as described above. Plasmids were then classified as “Conjugative,” “Mobilizable,” or “Non-mobilizable.”

### 2.4. Clustering putative BHR plasmids and determining their unique features

Average nucleotide identity (ANI) was calculated for each pair plasmid sequences, which were then clustered using an ANI threshold of 95% through the “ANIm” tool in PyANI v0.2.12 (Pritchard et al., 2016). Plasmid sequences were then annotated using prokka v1.14.5 to compare annotated vs. unannotated genes in each cluster (Seemann, 2014).

## 3. Results

### 3.1. Plasmid and ARG distribution in the CDC & FDA AR Isolate Bank

**Figure 1:**
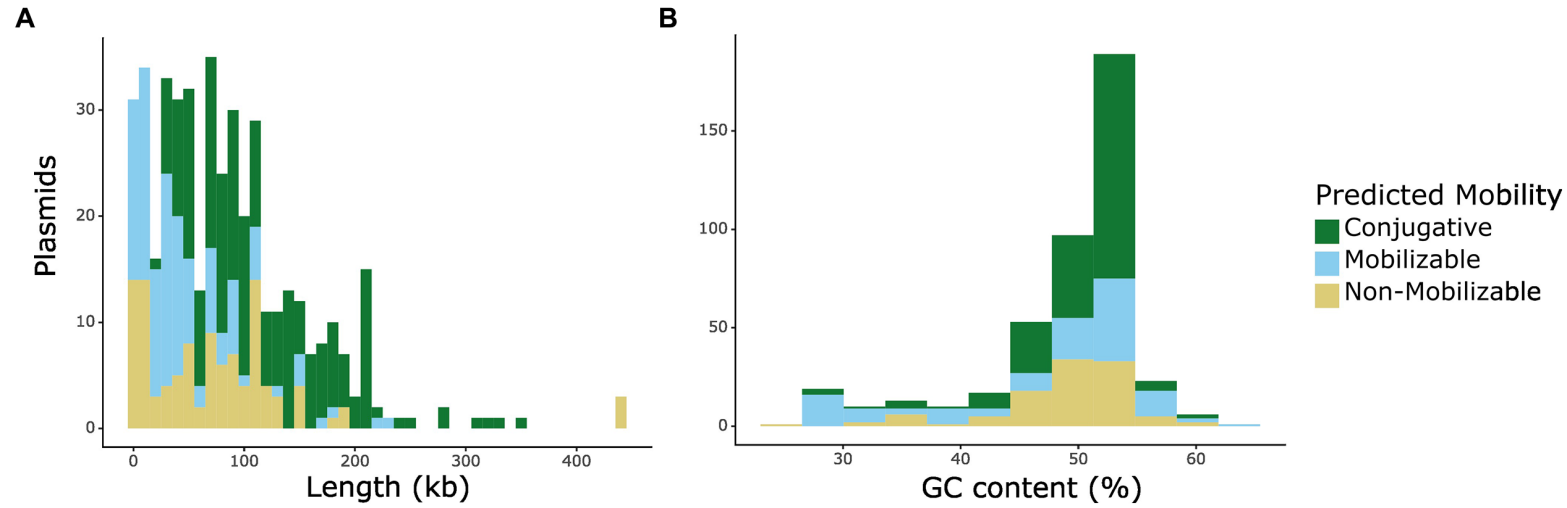
Mobility distribution of plasmids in isolates from the CDC & FDA AR Isolate Bank by **(A)** plasmid length and **(B)** GC content (%). Mobility is indicated by color: non-mobilizable plasmids are indicated by yellow, mobilizable plasmids are indicated by light blue, and conjugative plasmids are indicated by green.

We identified 439 putative plasmid sequences from 199 closed genomes. After searching for conjugation-related sequences, we found that 332 of these putative plasmids are predicted to be transferable through conjugation due to the presence of an origin of transfer. From the transferable plasmids, 206 are predicted to be conjugative, or self-transmissible, by encoding for a relaxase and T4SS proteins, while 126 are predicted to be mobilizable, containing an origin of transfer, but lacking T4SS machinery. Sequence length varied from 911 bp-439 kb, with a median length of 73.4 kb. We note that the shorter end of this range is more likely to be plasmid-derived sequences rather than complete plasmids themselves. Interestingly, we observed that mobilizable (mob) plasmids tend to be longer than non-mobilizable (non-mob) ones (Kruskal-Wallis test, followed by Dunn’s multiple comparison test, P_KW-D_ = 6.02×10^−4^), and that conjugative (conj) plasmids tend to be longer than those that are only mobilizable (P_KW-D_ = 5.57×10^−9^) (Figure 1A). These results are in accordance with previous data that showed a relationship between plasmid length and mobility (Smillie et al., 2010). It has been suggested that plasmid GC content is an important factor in determining host-range (Shintani et al., 2015). Therefore, we also analyzed the plasmid sequences’ GC content and found that, consistent with other reports (Shintani et al., 2015; Che et al., 2021), conjugative plasmids have comparably higher GC content than either mobilizable or non-mobilizable plasmids (mob P_KW-D_ = 0.034, non-mob P_KW-D_ = 0.029) (Figure 1B).

There were 135 unique antibiotic resistance genes in the plasmid sequences. We observed a relationship between presence or absence of an ARG and plasmid mobility (non-mob vs. mob P_KW-D_ = 2.83×10^−6^, non-mob vs. conj P_KW-D_ = 1.56×10^−17^), confirming previous results (Che et al., 2021; Newbury et al., 2022). The three ARGs with the most occurrences in the dataset were the sulfonamide resistance gene *sul1*, the antiseptic resistance gene *qacEdelta1*, and *TEM-1*, which encodes a beta-lactamase, with 104, 94, and 82 occurrences, respectively. Plasmids containing *TEM-1* were especially of note since 88.3% (68/77) of plasmids containing at least one copy of the gene were conjugative or mobilizable. Interestingly, genes that provide resistance to aminoglycosides were the most prevalent when grouped by antibiotic class (Supplementary Figure S1).

### 3.2. Classifying retrieved mobilizable and conjugative plasmids

**Figure 2:**
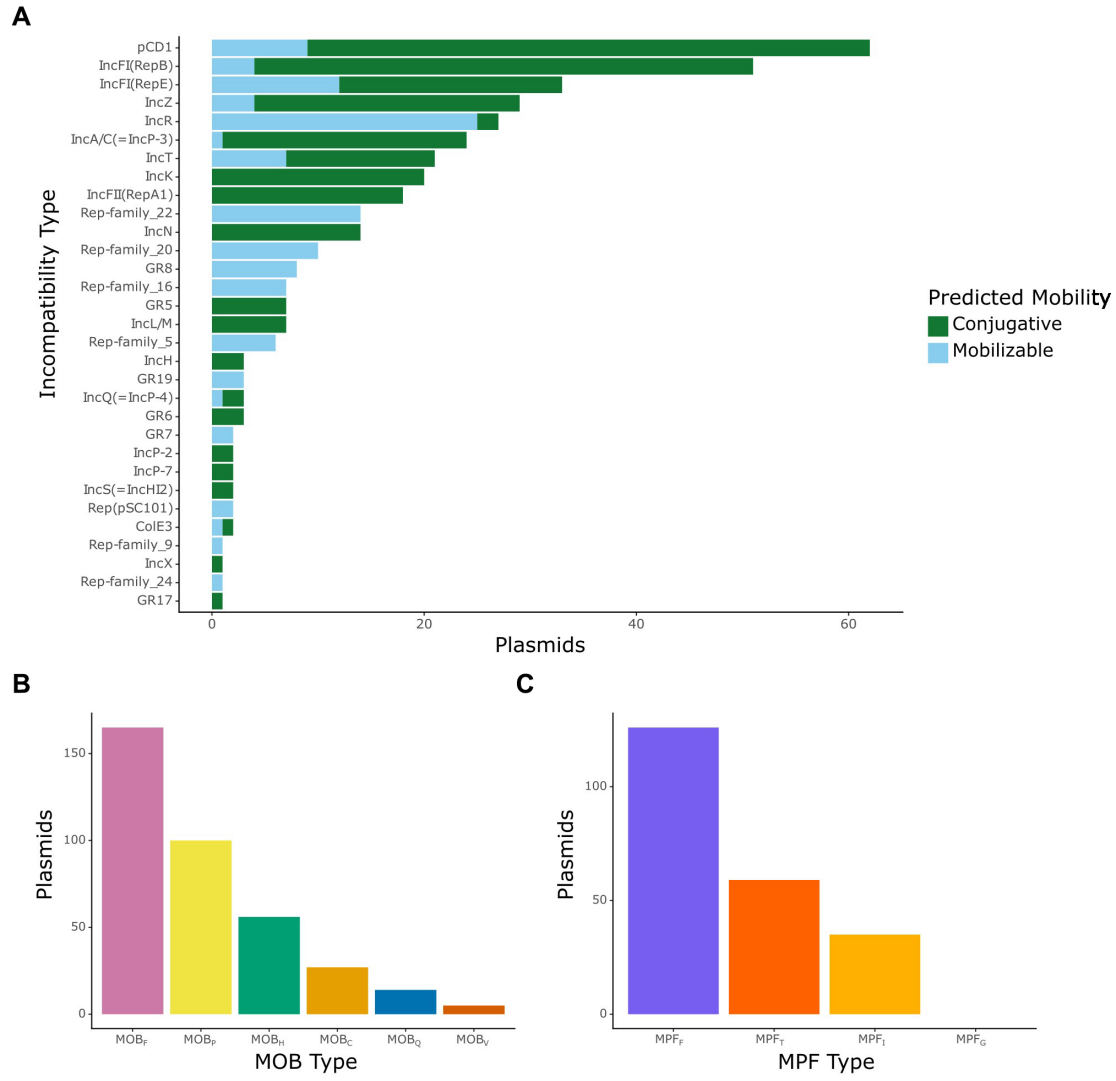
Classification of mobilizable and conjugative plasmids. **(A)** Number of plasmids by incompatibility type colored by their predicted mobility, where green indicates a conjugative plasmid and light blue indicates a mobilizable plasmid. **(B)** Number of plasmids by the mobilization (MOB) type of their encoded relaxases. **(C)** Number of plasmids by the mating-pair formation (MPF) type of their encoded T4SS genes.

After obtaining the distribution of plasmids and ARGs in the dataset, we focused on classifying the mobilizable and conjugative plasmids by incompatibility type, relaxase type (also known as MOB type), and T4SS type (also known as MPF type). Incompatibility (Inc) type varied within our dataset, with 239 mobilizable or conjugative plasmids being identified under one of 31 known Inc. types (Figure 2A). The most common Inc. type in our dataset was IncF, with 118 plasmids, which is consistent with previous data that have shown that IncF plasmids are commonly associated with extended spectrum beta-lactamases (Rozwandowicz et al., 2018).

MOB and MPF types are commonly used to classify mobilizable and conjugative plasmids based on similarity between their relaxase or T4SS proteins, respectively (Smillie et al., 2010). We identified relaxase gene sequences in 276 plasmids, with representation of all major MOB types (Figure 2B). MOB_F_ was the most common type of relaxase found with 120 plasmids containing this type. This type of relaxase is commonly associated with IncF plasmids (Rozwandowicz et al., 2018), so this result is not surprising. Similarly, we observed that the most common MPF type in the conjugative plasmids of this dataset was MPF_F_, with 123 plasmids encoding MPF_F_ T4SS proteins (Figure 2C). It should be noted that since our analysis did not include integrative conjugative elements in chromosomes, we did not observe any putative plasmid sequence containing MPF_G_ type T4SS proteins.

### 3.3. Analyzing plasmid host-range to choose potential BHR plasmids

**Figure 3:**
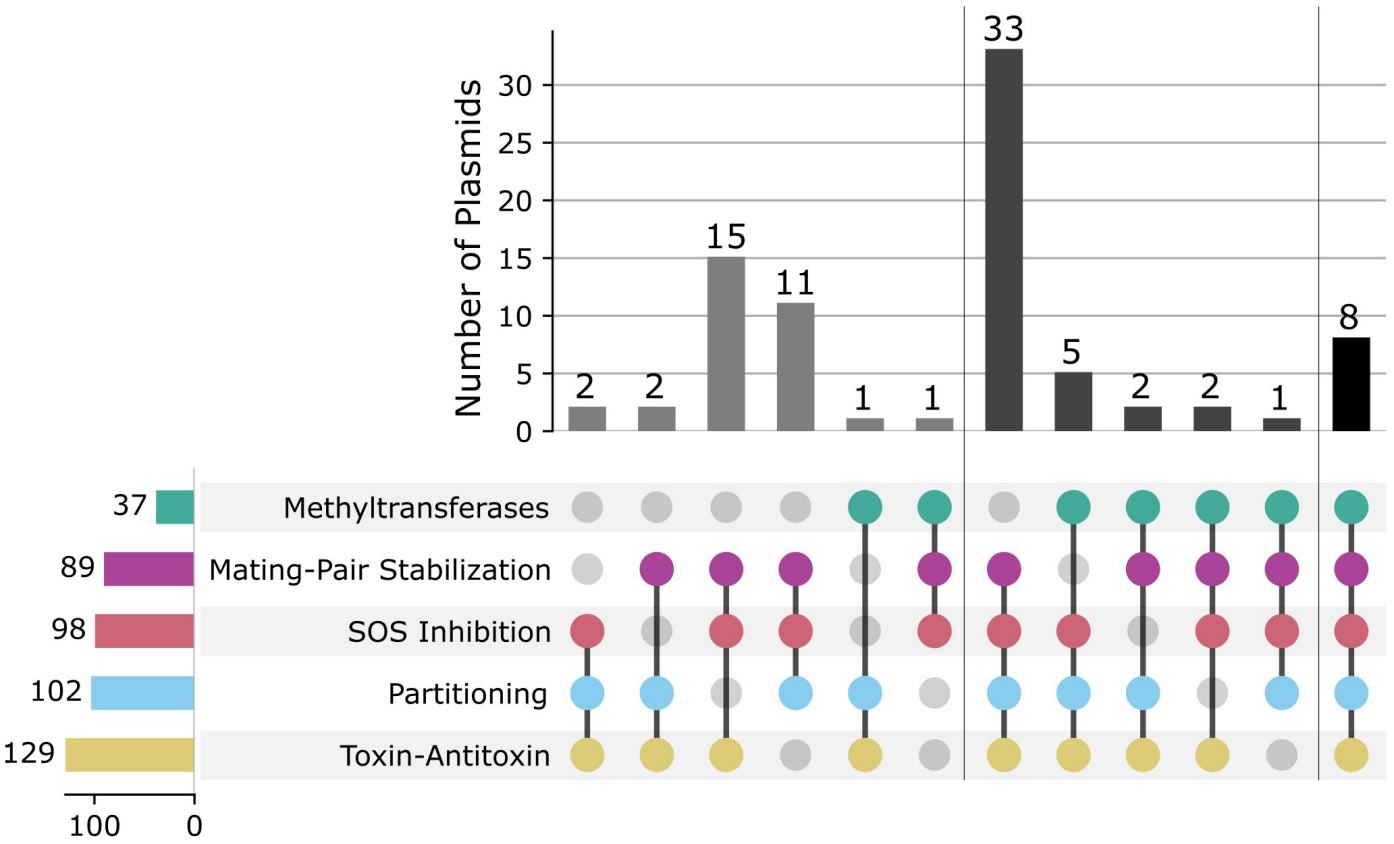
Analysis of host-range related genes. Groups are divided by presence or absence of at least 3 of the following: methyltransferase (green), mating-pair stabilization (purple), SOS inhibition (red), partitioning (light blue), and toxin-antitoxin (yellow) genes.

In order to identify candidate plasmids with broad host-ranges, we analyzed the presence of various proteins that can influence host-range in the mobilizable and conjugative plasmids: toxin-antitoxin systems (T-AT), partitioning proteins (Par), plasmid SOS inhibition (Psi), mating-pair stabilization (MPS), and methyltransferases (MTases) (Figure 3). T-AT systems, also known as post-segregational killing systems, are commonly found in mobile genetic elements and improve plasmid stability by ensuring that all daughter cells contain a copy of the plasmid after segregation (Jurėnas et al., 2022). Daughter cells that do not receive a copy of the plasmid are typically killed by the present toxin due to the lack of constant expression of its cognate antitoxin (Jurėnas et al., 2022). We found 167 plasmids encoding toxins from T-AT systems, but only 105 of these also encoded its antitoxin pair. It has been shown that toxins from T-AT systems can also be used as secreted effectors for virulence (Triplett et al., 2016), so it is not surprising to find plasmids that solely contain the toxin gene. The most common T-AT pair was *ccdA*:*ccdB*, first found in F plasmids (Bernard and Couturier, 1992). We then searched for partitioning proteins, which are known to aid in plasmid stability by directing plasmid DNA to daughter cells during cell division (Baxter and Funnell, 2014). We found 102 plasmids with Par genes, the most common of which was *sopB*, the F plasmid centromere-binding protein, occurring in 52 plasmids. Although plasmid SOS inhibition and mating-pair stabilization are two relatively less well-studied processes, with only a few experimentally verified proteins that perform each function, we decided to include them in our search due to their potential importance in determining host-range (Neil et al., 2021a). SOS inhibition prevents RecA binding to the single-stranded plasmid DNA when it is transferred to a recipient, while MPS prevents the interaction between donor and recipient to be interrupted during DNA transfer (Neil et al., 2021a). Surprisingly, we found 98 plasmids encoding Psi genes and 89 plasmids encoding MPS genes. The most common Psi gene was *psiB*, while the most common MPS gene was *traN*. Both of these proteins were first described in the F plasmid (Klimke et al., 2005; Pérez-Mendoza and de la Cruz, 2009). Finally, we decided to search for MTase genes in the plasmid sequences as a proxy for restriction avoidance, one of the ways plasmids evade host defenses during conjugation (Shaw et al., 2022). It has previously been shown that plasmids and other MGEs carry solitary MTases as a way to prevent restriction from recipient hosts (Oliveira et al., 2014). We found 37 plasmids encoding MTases from 14 different restriction-modification systems.

To obtain putative broad host range plasmids, we chose sequences that contained at least three of the five characteristics analyzed earlier (T-AT systems, Par proteins, Psi proteins, MPS proteins, and MTases), resulting in 82 potentially broad host-range plasmid sequences. To validate host-ranges for these putative BHR plasmids, we used MOB-suite’s prediction of “host-range rank” (Robertson et al., 2020) and found that all plasmids are predicted to transfer at the order-level or above, with 3 plasmid sequences predicted to transfer at the phylum-level (Supplementary Table S1). We clustered these candidate sequences by average nucleotide identity (ANI) and found that many sequences had (>95%) ANI and clustered together (Supplementary Figure S2). This resulted in 22 different candidate plasmid types (Supplementary Table S3). The presence of very similar plasmids in distinct isolates suggests previous transfer of these, confirming their capabilities as mobile delivery vectors.

### 3.4. Analyzing putative BHR plasmids gene content

In order to learn more about our putative BHR plasmids, we analyzed their gene content in search of virulence factor (VF) genes, a potential limitation to the use of these plasmids in animal communities. Our VF gene search showed that out of the 22 candidate plasmid types, 13 contained a plasmid that did not have known VF genes. We obtained 27 plasmid sequences that did not encode for a VF (Supplementary Table S3). Additionally, we annotated the 82 putative BHR plasmid sequences and determined the number of coding sequences that were labeled as ‘hypothetical protein’. This allowed us to determine how many features of the putative BHR plasmids were similar to those of previously characterized plasmids. The median number of features that could not be annotated reliably per plasmid was 57.35% (Supplementary Table S3), emphasizing that these plasmids potentially encode for proteins which we cannot predict function. These proteins could be related with maintenance of a broad host-range and are potential targets for further investigation.

## 4. Discussion

As microbiome engineering technology is optimized further, it is necessary to put a focus on the host-range of the delivery vectors that are used for genetic manipulation. This work presents a characterization of plasmids belonging to the CDC & FDA AR Isolate bank and a suite of putative BHR plasmids that could be used as vectors in engineering applications. Although they are all predicted to have broad host-ranges, the plasmids presented here vary in whether they are conjugative or mobilizable, their conjugation systems, and ARG distribution, offering users options when considering which plasmids to use. However, this work is limited by two factors: (1) the dataset used is overrepresented by *Enterobacteriaceae* isolates and (2) our current understanding of conjugation and the mechanisms that underlie host-range are limited to those from well-studied model plasmids mostly studied *in vitro* (Neil et al., 2021a). A further variety of conjugation systems and host-ranges could be obtained as we learn more about plasmid host-range in novel, non-model plasmids (Yu et al., 2022). Delivery of these plasmids to diverse microbial communities will be dependent on the members of each community and host-encoded factors that can affect these plasmids’ effective host-ranges (Neil et al., 2021a). Therefore, a better understanding of the hosts and plasmidomes associated with specific microbial communities will also provide clarification on the usefulness of individual plasmids for delivery to specific niches.

The putative vectors described here are predicted to have broad host-ranges through both an analysis of their presence in distinct phylogenetic groups and plasmid-encoded factors. Nonetheless, future work will be needed to characterize their host-ranges *in vitro* and *in situ*. These additional studies will not only further characterize these plasmids for use in microbiome engineering, but also add to our knowledge of ARG-carrying plasmid biology. As global antibiotic resistance in pathogens is rising (Murray et al., 2022), it is necessary to understand how ARG-carrying plasmids transfer through species. However, the presence of ARGs in these plasmids does provide a challenge for their use in microbiome engineering, due to the risk of antibiotic resistance acquisition by members of the community. It is also necessary to note that many of the plasmids are larger than 50 kb, which could be an additional limitation for their use. Further optimization could be made by eliminating ARGs from these plasmids or engineering smaller, mobilizable vectors from those described in this work. A starting general workflow for those who would like to use these plasmids is presented in Supplementary Figure S3. It is important to note that each project will involve a high level of customization to the user’s need.

Finally, these plasmids provide a starting point for further development of microbiome engineering systems based on conjugation. Due to their broad-host nature, they can be used to target genes present in multiple species throughout the microbiome, such as the cardiovascular disease-associated *cutC/cutD* genes (Koeth et al., 2013; Rath et al., 2017). Therefore, by using conjugative broad-host delivery vectors to perform targeted genetic manipulations in the human microbiome, it would be possible to eliminate potentially deleterious functions from the community and further our understanding of the host–microbe interactions that underlie disease.

## Data availability statement

The dataset analyzed for this study can be found in the NCBI database under BioProject accession PRJNA294416 (https://www.ncbi.nlm.nih.gov/bioproject/294416). Code used for analysis is available at https://github.com/hecgloyo/Characterizing_AR_Isolate_Plasmids.git.

## Author contributions

HL performed all analyses and wrote the first draft of the manuscript. HL and IB contributed to the conception and design of the study, wrote sections of the manuscript, and revised, read, and approved the submitted version.

## Funding

Funding for this study was provided by the National Institutes of Health (1DP2HL141007) and the Pew Charitable Trusts, Pew Biomedical Scholars Program (to IB).

## Conflict of interest

The authors declare that the research was conducted in the absence of any commercial or financial relationships that could be construed as a potential conflict of interest.

## Publisher’s note

All claims expressed in this article are solely those of the authors and do not necessarily represent those of their affiliated organizations, or those of the publisher, the editors and the reviewers. Any product that may be evaluated in this article, or claim that may be made by its manufacturer, is not guaranteed or endorsed by the publisher.
