# Supplementary material for: Characterizing conjugative plasmids from an antibiotic-resistant dataset for use as broad-host delivery vectors
Source: Front Microbiol. 2023 Jun 14;14:1199640. doi: 10.3389/fmicb.2023.1199640 (PMC10301749; doi:10.3389/fmicb.2023.1199640)
Supplement: Supplementary file 4 [file Data_Sheet_1.PDF]

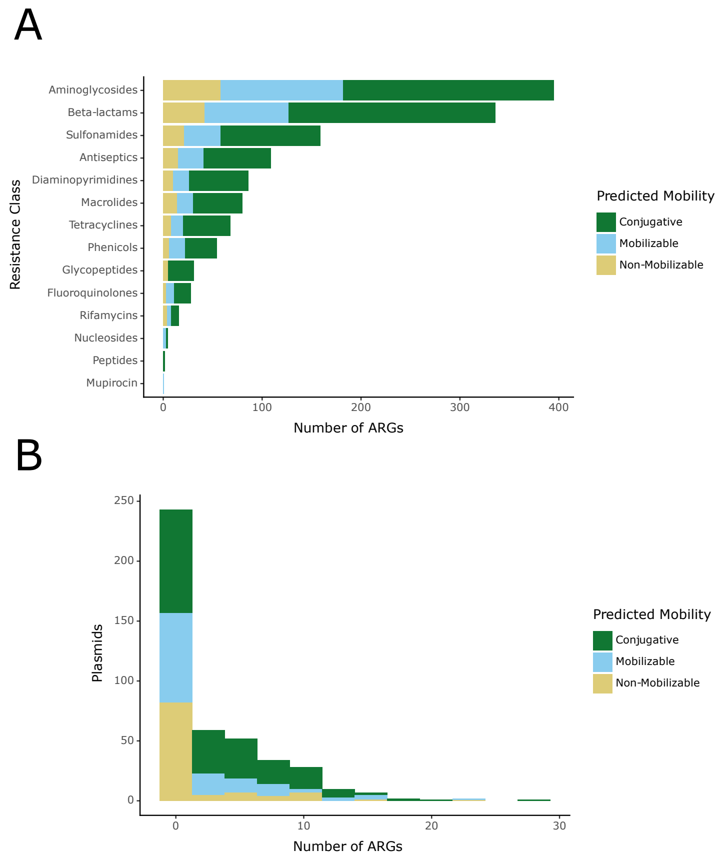

Supplementary Figure 1. Distribution of ARGs in CDC & FDA AR Isolate Bank plasmids. (A) Length of bars represents the number of occurrences of a gene in each resistance class. (B) The number of ARGs per plasmid. In both (A) and (B), color corresponds to the mobility of the plasmid it was observed in. Green indicates a conjugative plasmid, light blue indicates a mobilizable plasmid, and yellow indicates a non-mobilizable plasmid.

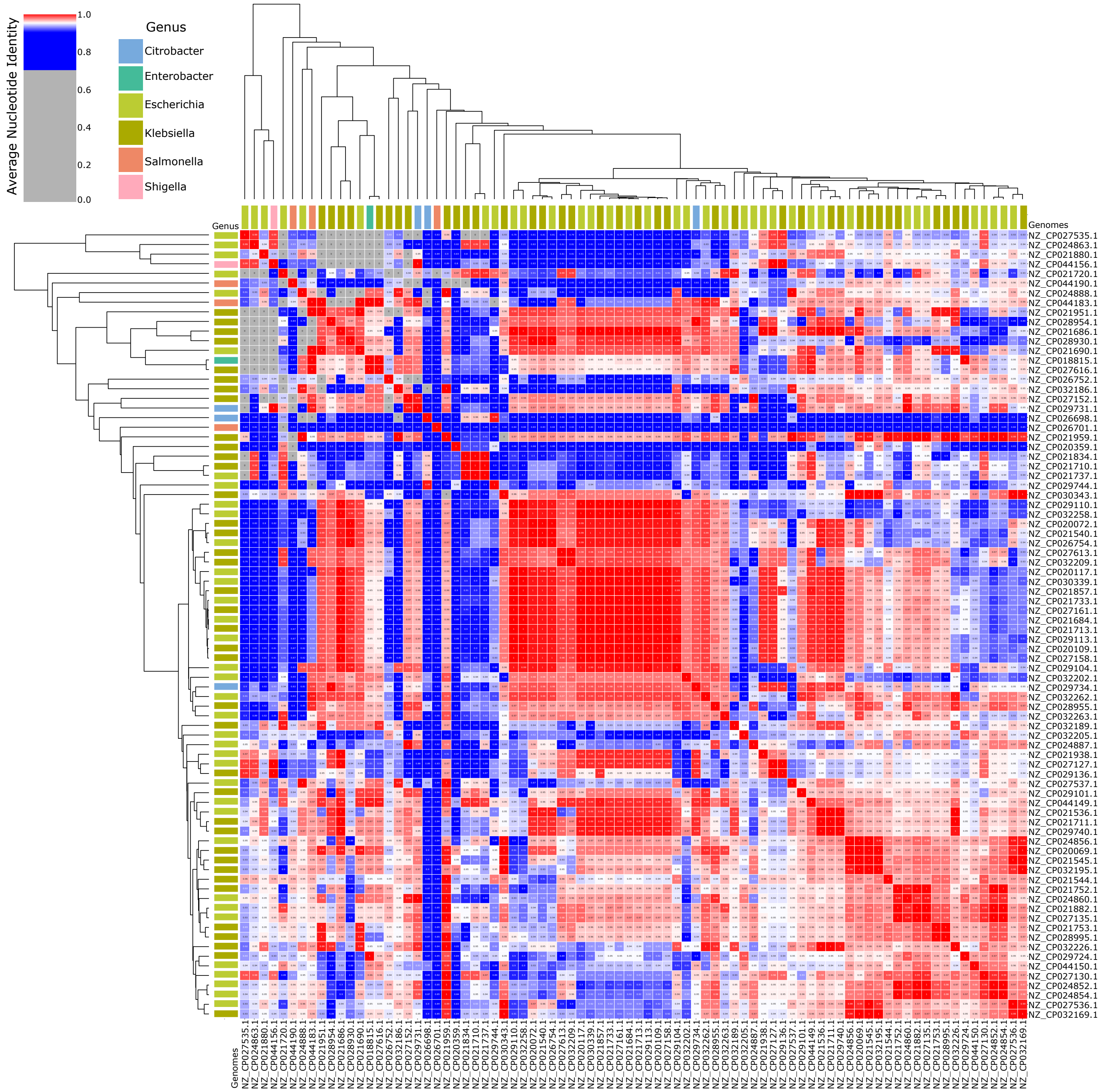

Supplementary Figure 2. Average Nucleotide Identity matrix for putative BHR plasmid sequences. Plasmids are identified by their NCBI Accession number. Plasmids that have an ANI over 0.95 are shaded in red, while those that do not meet this threshold are shaded in blue. Color intensity indicates closeness to the maximum (for red) or minimum (for blue). The genus of each plasmid's host is indicated by the color bar on the side of the matrix.

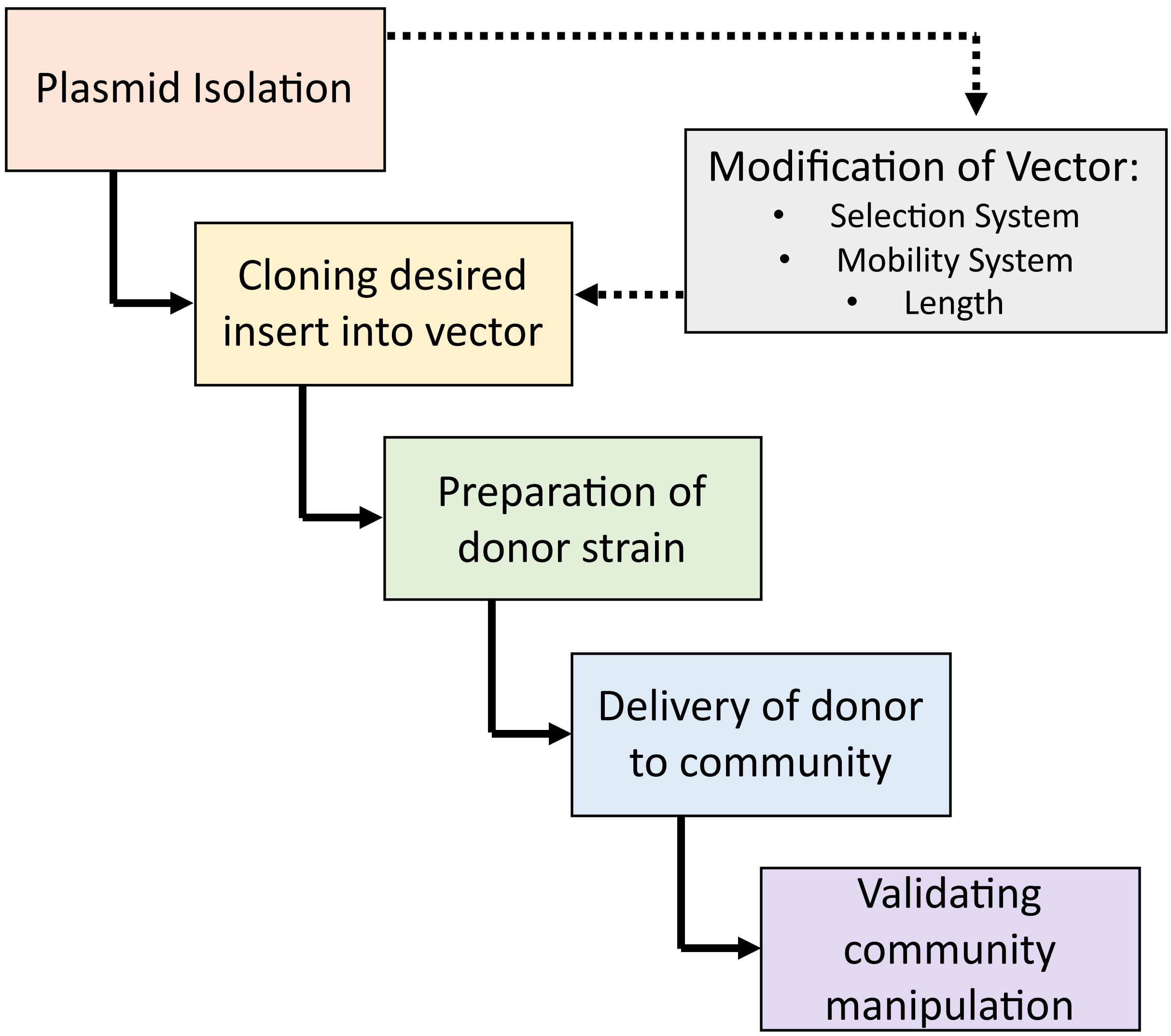

Supplementary Figure 3. Proposed workflow for use of BHR plasmids obtained from CDC & FDA AR Isolate Bank. The "Modification of Vector" step is optional, but recommended.
